# Docol 1300M Micro-Jet-Cooled Weld in Microstructural and Mechanical Approaches concerning Applications at Cyclic Loading

**DOI:** 10.3390/ma17122934

**Published:** 2024-06-15

**Authors:** Tomasz Węgrzyn, Klaudiusz Gołombek, Bożena Szczucka-Lasota, Tadeusz Szymczak, Bogusław Łazarz, Krzysztof Lukaszkowicz

**Affiliations:** 1 Faculty of Transport and Aviation Engineering, Silesian University of Technology, Krasinskiego 8, 40-019 Katowice, Poland; tomasz.wegrzyn@polsl.pl (T.W.); bozena.szczucka-lasota@polsl.pl (B.S.-L.); boguslaw.lazarz@polsl.pl (B.Ł.); 2 Faculty of Mechanical Engineering, Silesian University of Technology, Konarskiego 18A, 44-100 Gliwice, Poland; klaudiusz.golombek@polsl.pl; 3 Department of Vehicle Type-Approval & Testing, Motor Transport Institute, Jagiellońska 80, 03-301 Warsaw, Poland

**Keywords:** high-strength steel, Docol, micro-jet cooling, welding, mechanical resistance, microstructure, mini-specimen, fatigue

## Abstract

The application of advanced high-strength steel grades (AHSS) in different kinds of industry is connected to more than their attractive mechanical properties. The present paper focuses on improving the welding Docol 1300M steel to reach an acceptable microstructure and mechanical parameters. It was decided to manufacture joints with different welding parameters using different filler materials. The electrode wires were varied to increase the carbon content in the weld, and nitrogen was added to the argon shielding mixture to obtain non-metallic inclusions that strengthen the fusion zone. Specimens of joints welded with the gas metal arc welding (GMAW) process for non-destructive and destructive tests were examined. Tensile and bending tests as well as microscopic inspections using a light (LM) and scanning electron microscope (SEM) were also conducted. The results from the fatigue test confirmed the validity of the proposed welding process for the Docol 1300M joint. The collected data enabled the following conclusion: The article’s novelty is represented by the use of shielding gas mixtures containing argon and nitrogen in the GMAW welding process of AHSS steel to create titanium non-metallic inclusions, which will translate into better performance properties of the entire joint.

## 1. Introduction

The manufacturing of modern components requires advanced high-strength steel (AHSS) grades concerning its attractive mechanical features compared to the other applied structural materials [1,2,3], such as a high value of ultimate tensile strength and good fatigue properties [4,5,6,7,8,9,10]. The use of this kind of steel enables obtaining a significant reduction of weight, which is very important, especially for different branches of industry, i.e., automotive for manufacturing at trailer and semi-trailer and safety zones, roofs, sills, bumpers, and seat structures [11,12,13,14] as well as marine, concerning container types production and road transport if reduction of operation loading is considered [15,16,17,18,19]. Nevertheless, it is worth emphasizing that the difference in the mechanical properties of the base material and the weld of high-strength steel grades may often lead to cracks occurrence in the heat-affected zone (HAZ) [14,17]. This means the component with damages may not be indicated as the correct one for application, and it should be redesigned as well, and the joining process should be significantly changed in the stages concerning its parameters [20]. This approach is crucial from the engineering point of view because this enables the avoidance of catastrophic accidents and can preserve the health or life of many people [21,22]. This shows that welding AHSS and UHSS is difficult. The meaning of this stage is also indicated by steel manufacturers that have proposed guidelines for welding groups [13,23,24]. This is very restricted, and any deviations from the requirements are not allowed. Taking this into account, it is justified to assert that although high-strength steel grades are very attractive for their high-level mechanical parameters, they require a very suited technology for joint types manufacturing, for its quality, and for covering a lot of applications and various operational conditions, such as static, dynamic, and cyclic.

Thicker sheets are recommended to be welded with preheating and controlled linear energy. Thinner sheets, especially single-pass joints, can be welded using a forming pad without preheating. High Ti content is used in AHSS steels, which exceeds the Ti content in low-alloyed steels by more than 10 times. Therefore, various types of non-metallic inclusions are formed in the material, mainly TiC carbides, TiN nitrides, TiO oxides, and Ti(N,C) carbonitrides [25,26].

The addition of Ti in these steels is justified because the titanium inclusions increase the tensile strength. Despite the high content of Ti in the base material, the ultimate tensile strength of the joint is worse than that of the base material. In one study, it was decided to make test joints to check the possibility of increasing the strength of the joint and bringing the strength of the joint closer to the strength of the base material. It was decided to increase the carbon content in the weld by using an electrode wire with a higher carbon content, and it was decided to increase the nitrogen content in the shielding gas mixture. This treatment was aimed at increasing the strength of the joint due to the presence of precipitates of the types TiC, TiN, and TiNC [27,28]. It was found that classic welding processes need to be modified to achieve better results [4,5,20,21,22]. Because high-strength steel grades are needed in the heavy industry, an attempt was made to modify the classic welding process (MAG, metal active gas).

Some authors, based on experience with Docol-grade (1200M) steel welding [29], have suggested that this kind of material is not suitably weldable because the mechanical properties of the joint and the base material are very different. Authors working with HSS steels [25,27] have come to similar conclusions. They have found that a careful thermodynamic analysis of the process (preheating, current–voltage parameters, and welding speed) should be carried out for a properly made joint. They have indicated that poorly selected welding parameters will translate into cracks in the HAZ. It is worth expressing that carbon is the main factor influencing the weldability of the AHSS [11,14]. In the manuscript [30], T. Vuherer et al. presented the results of an investigation on a martensitic coarse grain heat-affected zone (HAZ) in welded joints. The authors analyzed the mechanical properties and microstructure of the HAZ. The presented results confirmed that the HAZ microstructure consists of lath martensite. Micro-jet cooling may play an important role in the welding of unalloyed steels as well as high-strength steels. This affects the thermodynamic conditions of the welding process. Micro-jet cooling affects the nature of phase changes and the formation and growth of various non-metallic inclusions, which determine the strength of the joint. This joining method enables control of the microstructure of the welds. This innovative welding process was realized successfully, mainly in welding low-alloy materials [20]. Other authors indicated the adverse effects of hydrogen when welding high-strength steels and hydrogen reducing. For this case, preheating was proposed. Other authors found that accumulating excessive amounts of hydrogen leads to the formation of hydrogen-induced cracking (HIC) [31]. This cracking in high-strength steels is mainly observed at the grain boundaries and in solvent contact with non-metallic inclusions [32]. During the welding of Docol steel, the influence of nitrogen in the GMAW argon shielding mixtures was not considered. This is a research gap and one of the reasons for undertaking this topic in the present paper.

For this approach, attention has been paid not only to the metallurgical aspect but also to the thermodynamic one. It was decided to make welded joints in a shielding gas mixture containing argon and nitrogen. So far, AHSS steels have been welded mainly in shielding mixtures containing Ar-CO_2_ and Ar-O_2_. These mixtures allowed for a fairly correct execution of the joint. The addition of oxygen or CO_2_ in argon mixtures was primarily intended to cause better penetration and provide better geometry of the welds [16,20]. The influence of active elements forming non-metallic inclusions in AHSS steels and the role of non-metallic inclusions in AHSS steels on joint properties are not widely presented in the literature.

The paper aims to find technology for welding Docol 1300M steel to obtain a higher value of ultimate tensile strength of the joint obtained in previous processes. It was decided to follow this idea through metallurgical treatments (increasing the content of carbon and nitrogen in the weld metal, which translates into the formation of various titanium inclusions, which can strengthen the joint). First of all, it was expected to obtain non-metallic titanium inclusions (considering the fact that the AHSS steel contains 10 times more Ti than low-alloy steels) such as TiC, which could strengthen the solution, and TiO and TiC, in contact with which fine-grained ferrite can easily nucleate. This will prevent the growth of ferrite, which is recommended in AHSS steels [29,33,34,35].

## 2. Materials and Methods

**Figure 1:**
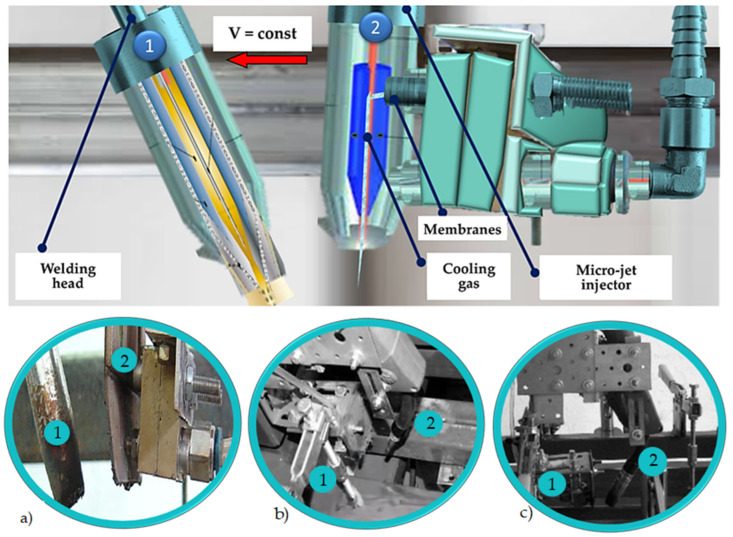
Welding head and micro-jet injector: (**a**) that recommended for AHSS steel; (**b**,**c**) examples for other materials.

The welding process (Figure 1) was directly used for specimens manufacturing in view of the following details:

- A welded (BW) butt weld of Docol 1300M steel plate with a thickness of 1.8 mm was prepared;
- The GMAW process with micro-jet cooling in the lower position (PA) was chosen according to the requirements of the PN-EN ISO 15614-1:2017-08 standards [36];
- The gap for the joint was represented by a thin rectangle at a thickness of 0 ± 2 mm;
- The specimen’s total dimensions are expressed by the following values: 1.8 mm × 250 mm × 400 mm.

The welding was carried out on the stage presented in Figure 1a with the following parameters of micro-jet cooling: micro-jet gas—argon; gas pressure—0.55 MPa; diameter of the stream—0.75 µm.

It was decided to produce specimen welds using the GMAW process, varying shielding gas mixtures and electrode wires. Three shielding gases were chosen: pure Ar, Ar-1% N_2_, and Ar-2% N_2_ (according to the PN-EN 14175:2009 standard [37]). The specimens were welded with two electrode wires (at the requirements of the PN-EN ISO 16834:2012 standard [38]). Table 1 shows the chemical composition of the tested Docol 1300M steel, while Table 2 illustrates the chemical composition of both electrode wires. It is rather similar, although they differ slightly in the content of two important elements (C and Ti).

Union X96 filler wire has a more significant proportion of both elements. Another important observation is that the Ti content in both filler wires is at least twice as high as standard low-alloy steel. This fact can translate into the formation of various titanium inclusions, such as TiN and Ti(C,N). The welding parameters are summarized in Table 3.

The following non-destructive tests (NDT) were used for the specimen selection:

- Visual tests (VT) of welded joints according to the PN-EN ISO 17638:2017-01 standard [39] and the assessment criteria following to the PN-EN ISO 5817:2023-08 requirements [40];
- Magnetic-powder tests (MT) of welded joints according to the PN-EN ISO 17638 standard and the assessment criteria according to PN-EN ISO 5817:2023-08 [40] using a magnetic flaw detector device type REM—230.

**Figure 2:**
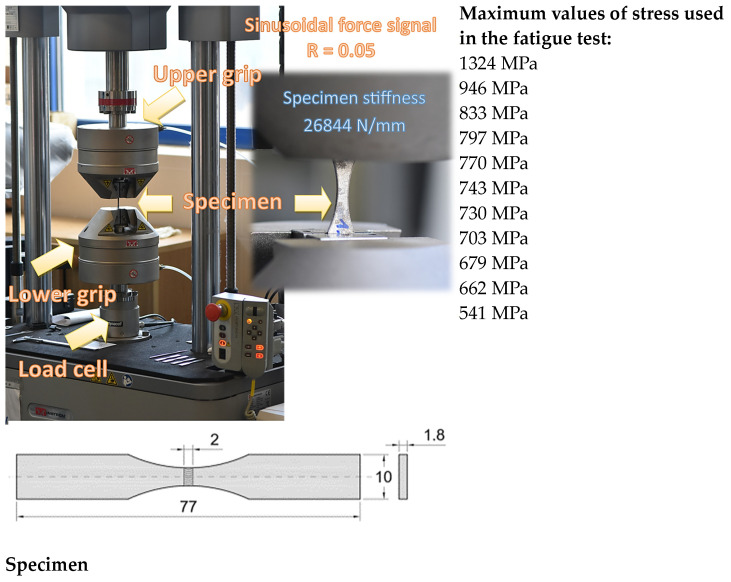

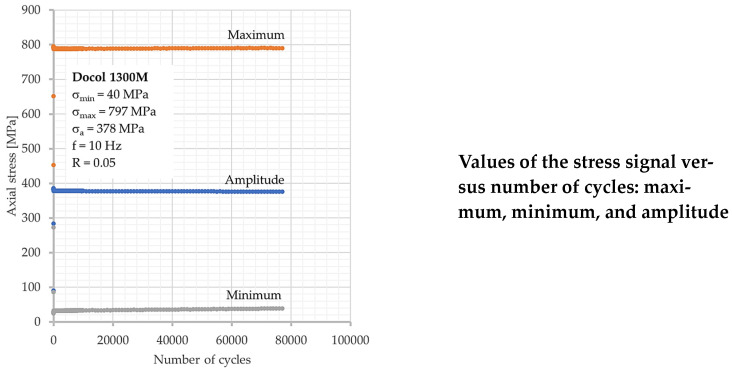
E10000 Electropuls Instron testing machine, specimen (with stiffness = 26,844 N/mm) and the stress trends, maximum values of the stress used in the fatigue test, and specimen thickness = 1.80 mm.

In the second section of the experimental procedure, destructive tests were carried out:

- Visual tests on macro specimens (transverse specimens) of welded joints were made with the eye fitted with a magnifying glass at 3× magnification—tests were performed according to PN-EN ISO 17638 standard [39] with the test reagents according to PN-CR 12361 standard [41] and the assessment criteria according to PN-EN ISO 5817:2023-08 [40] standard;
- Observations of the specimen microstructure etched with the Nital reagent using a light microscope (LM) (Zeiss Axio Observer.Z1m, Manufacturer: Carl Zeiss Microscopy GmbH, Jena, Germany) and microstructure investigations were carried out using a high-resolution scanning electron microscope, Zeiss Supra 35 (Zeiss Supra 35, Manufacture: Carl Zeiss NTS GmbH, Oberkochen, Germany) with an accelerating voltage of 20 kV and magnifications of 70–15,000×. A secondary electron detector SE and backscatter electron detector BSE were used for the study. Analyses of chemical composition in micro-areas were performed using the EDX detector (Thermo Scientific™, EDX detector: Thermo Fisher Scientific, Waltham, MA, USA) with Pathfinder software (thermofisher.com) (EDX detector: Thermo Fisher Scientific, Waltham, MA, USA). This stage was performed at the EBSD camera (Orientation Imaging Microscopy v5 Analysis software version 5.31) and OIM Analysis software from EDAX;
- Tensile test of the welded specimens according to the PN-EN ISO 6892-1:2020 standard [42];
- Hardness test according to the PN-EN ISO 9015-1:2011 [43] and PN-EN ISO 6507-1:2018-05 [44] standards;
- Fatigue test on the collected specimen design was performed according to the rules of the ASTM E468-18 standard [45]. The fatigue test was conducted at room temperature, at stress ratio R = 0.05, f = 10 Hz, and sinusoidal stress cyclic signal for controlling the E10000 Instron electro-dynamic testing machine. The maximum values of axial stress used in the experiment were represented by 11 its levels (Figure 2). Maximum, minimum, and amplitude for the stress signal at 797 MPa are shown in Figure 2 as well. Due to the small size of the measurement gauge of 10 mm, the use of the extensometer was not possible because there was a high probability of its permanent failure. This means the hysteresis loops were not collected. Therefore, the main result of the fatigue test is expressed by the number of cycles to fracture and the Wöhler curve as well as fatigue fracture regions obtained by the macro-photography technique. The specimen shape and the testing machine are illustrated in Figure 2.

## 3. Results and Discussion

The joints obtained with different shielding gas mixtures with various nitrogen (N_2_) content (0%, 1%, and 2%) were tested. The results of the non-destructive testing of the joints produced with the use of various welding parameters are presented in Table 4.

The table analysis shows that the high nitrogen content in the gas mixture is unfavorable. Minor cracks in the joint were observed (about three cracks 2–3 mm long). For further tests, it was decided to take only those specimens that did not have welding defects and non-conformities found in the NDT (PG1, PG2, PG4, and PG5). The next part of the research was the hardness test. Only those specimens with no defects after NDT were taken for examination. Durability studies focused on the following areas of the joint: HAZ (heat-affected zone), weld, and base material. The test results are presented in Table 5. They are shown at values of Vickers, Brinell, and ultimate tensile strength (UTS) calculated based on the following relationship: UTS = 3.44 HB (based on the Brinell hardness results (HB) converted using the Vickers hardness values). The coefficient value equal to 3.44 was captured from ASTM 370 [46] using the proportion between values of Brinell hardness and ultimate tensile strength ranging from 371 MPa to 271 MPa and 1250 MPa to 900 MPa, respectively. Concerning difficulties in preparing tensile specimens based on the joint regions because the sections of the weld are very small, the values of Brinell hardness were also used for approaching the values of ultimate tensile strength, shown in Table 5. As can be seen, values of Brinell hardness may be easily used for assessing values of ultimate tensile strength. Therefore, the following conclusion was formulated: The Brinell hardness supports the results of tensile tests. Moreover, it is worth emphasizing that the Vickers and Brinell hardness methods are very often used by technicians and engineers in quality control. Nevertheless, crucial advantages and disadvantages of the employed hardness methods for examining the weld and its region can be indicated:

(a) Vickers hardness test

- Advantages: This method may be used for small regions, and an indent size is small;
- Disadvantages: The test zone should be polished, and there is an influence of the non-horizontal location of the specimen on the measurement results.

(b) Brinell hardness test

- Advantages: The specimen surface does not need to be polished, and there is negligible influence of non-horizontal specimen location on the result;
- Disadvantages: There is no possibility of use at small material zones, and the results depend on the load value.

The joint hardness analysis showed that the HAZ (heat-affected zone) and the base material have the highest hardness values. In contrast, the weld hardness is noticeably lower, regardless of the phenomenon of Docol 1300M steel recurrence after plastic processing, which occurred during thermal changes caused by welding energy. Increasing nitrogen in the weld (the effect of the shielding mixture) and carbon (the impact of the electrode wire leads to an increase in the hardness of the weld from 310 MPa (HV) to 325 MPa (HV)) were also noticed. Minor differences can be noticed in comparing results for UTS values of base metal with those captured in the tensile test (Table 6). This means the quality of the joint concerning fundamental the mechanical parameters is close to the results for the base metal. Moreover, the data presented in Table 6 may be used for the following engineering and scientific approaches: (A) designing/modelling at an elastic state (using Young’s modulus and/or proportional and elastic limits); (B) following the stage for the first plastic deformation (applying additionally yield stress); and (C) assuming the mechanical fatigue limit (applying ultimate tensile strength).

It is worth noticing that the yield stress and ultimate tensile strength are important mechanical parameters for the automotive industry because this kind of data is very often used to characterize a vehicle structure concerning the material used [11,23].

It was noted that the mechanical properties of the tested joints gave positive results. With the increase in carbon and nitrogen content in the weld metal, the strength of the joint and its yield point also increased. The best results were obtained for the connector with the sample PG5, which was made as follows:

- With a higher carbon content than PG1 and PG2;
- With a higher nitrogen content than PG4 filler wire.

**Figure 3:**
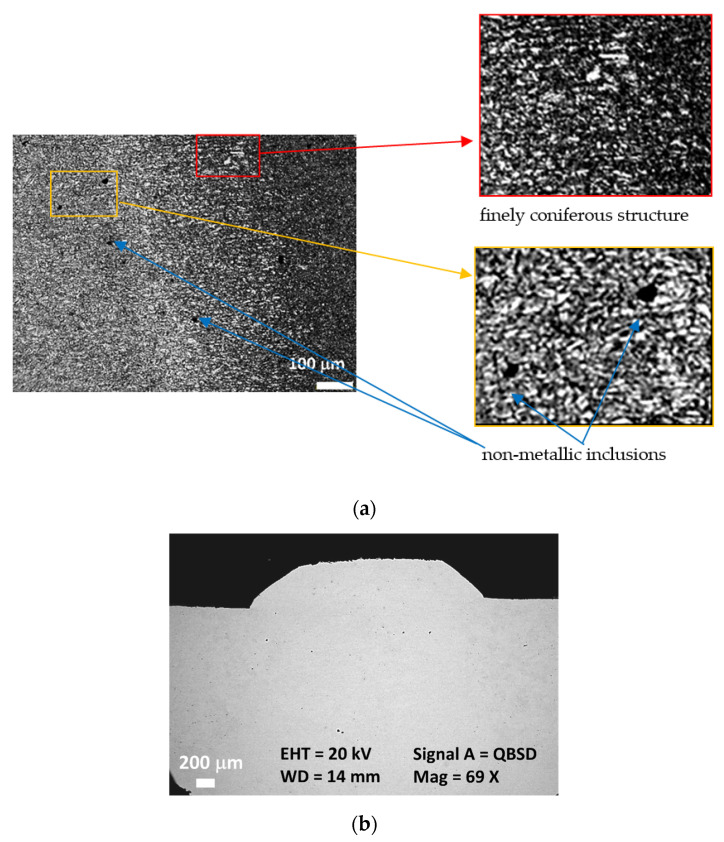
SPG5 joint: (**a**) near the weld fusion line, microstructure with non-metallic inclusions; (**b**) connect the cross-section with the visible area of the weld and heat-affected zones.

In further research, it was decided to check the microstructure, carry out observations under a scanning microscope, and perform fatigue tests for the PG5 joint. Figure 3 shows the microstructure of the heat-affected zone and weld for the PG5 sample.

We can thus observe the dominant finely coniferous structure (martensitic and ferrite). The non-metallic inclusions are shown by the black points in Figure 3a.

**Figure 4:**
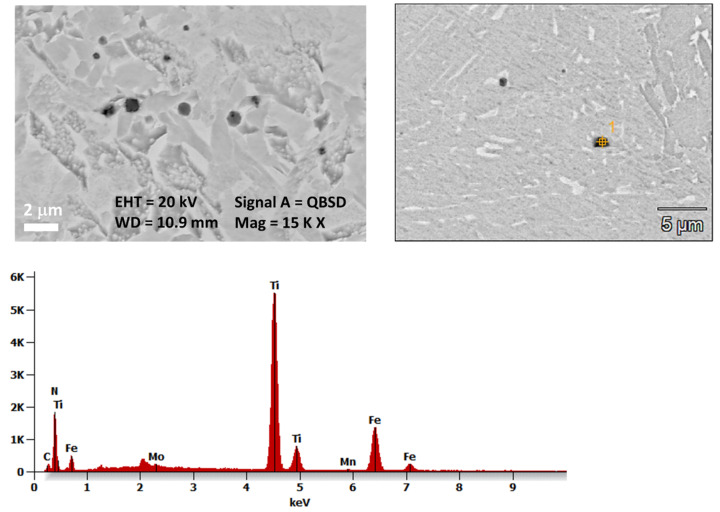
The microstructure of the Docol 1300M steel’s joint with visible non-metallic inclusions rich in carbon, nitrogen, and titanium, along with a point analysis of the EDX chemical composition.

**Figure 5:**
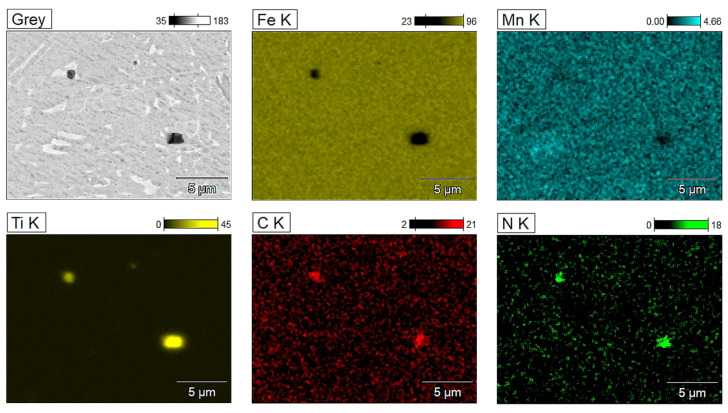
Elements distribution in the joint microstructure of the Docol 1300M steel.

Based on the feature of non-metallic inclusions, it can be assumed that they are nitrides and carbonitrides of titanium. To analyze the microstructure of the joint more precisely and to identify inclusions strengthening, SEM observations were applied. These results are shown in Figure 4 and Figure 5.

Based on SEM observation and analysis of the spectral spectrum, it was found that non-metallic inclusions in the joint consist of such elements as Ti, C, and N, which may correspond to carbonitrides of the Ti(C,N) type and Fe, Nb, and Mn. To confirm the presence of titanium carbonitrides in the microstructure, the tested joints were also tested using the EDX spectrometer, which shows the surface distribution of elements, as shown in Figure 5. It was found that the irregular non-metallic inclusions visible in the microstructure, with sizes in the range of 0.5–2 µm, and they are rich in titanium, nitrogen, and carbon.

**Figure 6:**
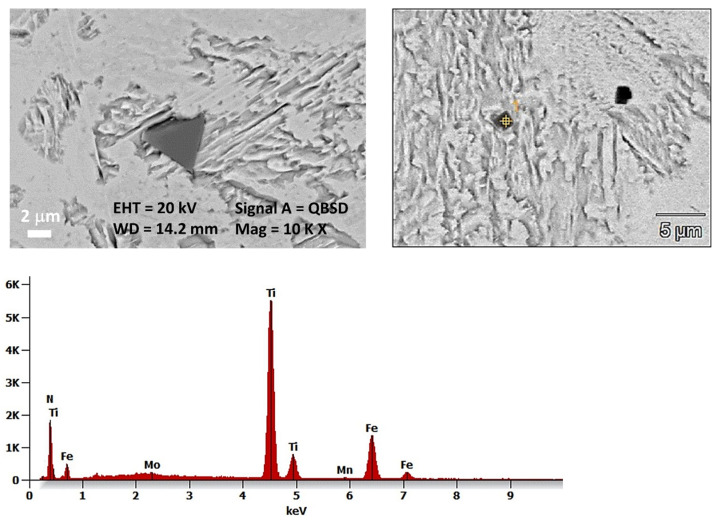
Elements of the microstructure of the joint for Docol 1300M steel with visible TiN-type non-metallic inclusions along with point analysis of the chemical composition of EDX.

**Figure 7:**
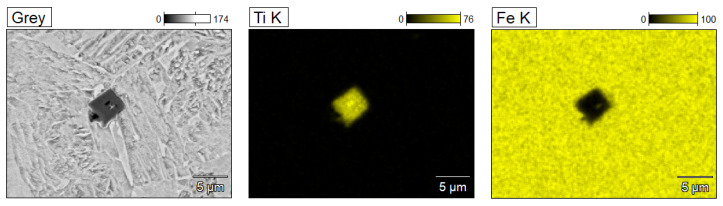

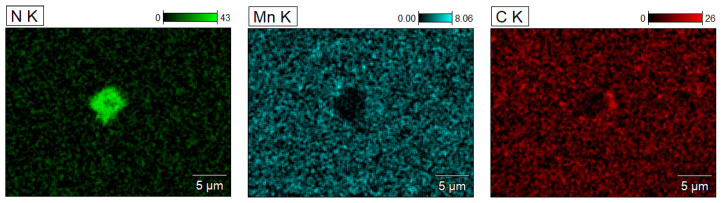
Elements distribution in the joint microstructure of the Docol 1300M steel.

In the joint, titanium nitrides with characteristic typical morphology were also observed, as shown in Figure 6. To check the presence of titanium nitrides in the joint microstructure, the surface distribution of elements was performed using the EDX spectrometer, shown in Figure 7. From the analysis of the spectral spectrum, it can be assumed that the present non-metallic inclusions in the joint correspond to nitrides of the TiN type with a simple crystal lattice (NaCl type). The size of titanium nitrides ranges from 0.5 to 4 µm.

**Figure 8:**
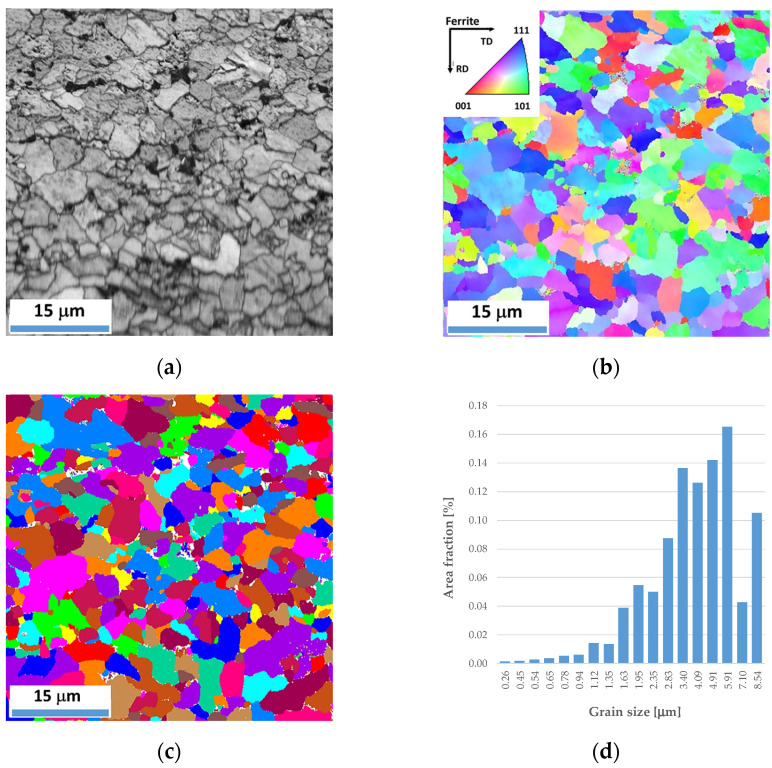
Microstructure of the base material of Docol 1300M: (**a**) SEM image, (**b**) inverse pole figure map (IPF), (**c**) image quality, and (**d**) distribution of grain size, EBSD.

**Figure 9:**
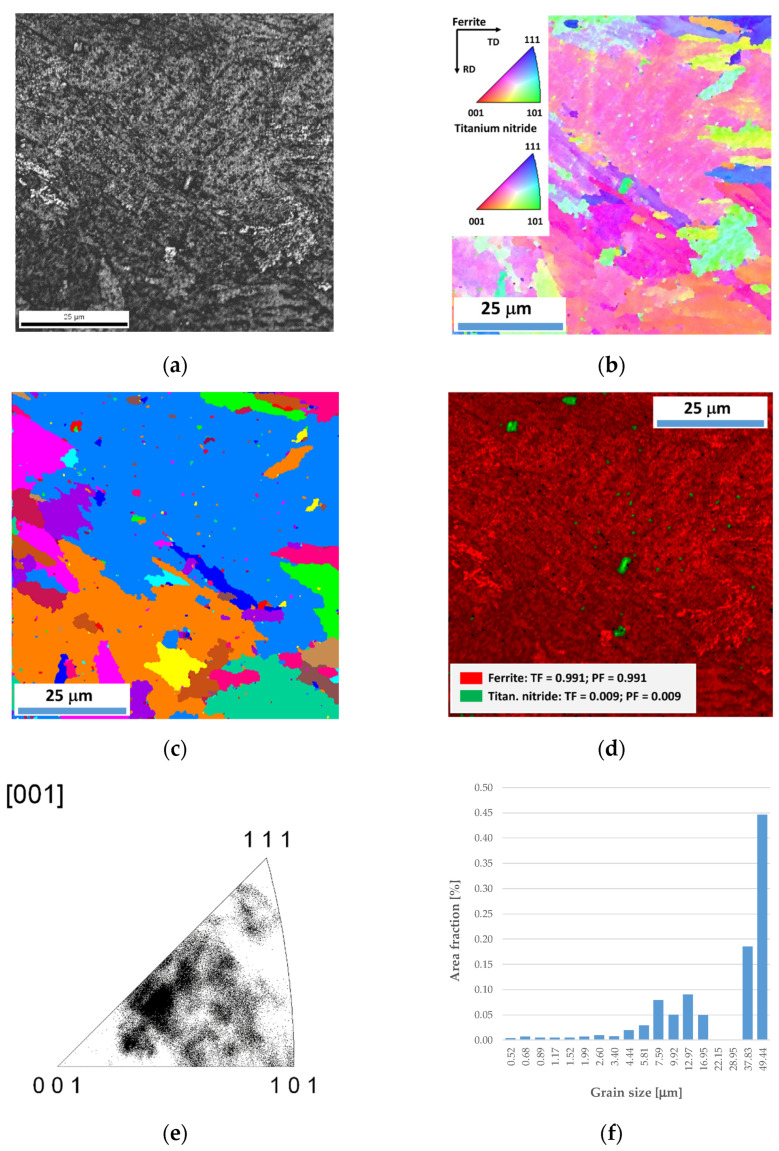
Microstructure of the welded material of Docol 1300M: (**a**) SEM image, (**b**) inverse pole figure map (IPF), (**c**) image of grain size, (**d**) corresponding phase map + IQ, (**e**) inverse pole figure, and (**f**) distribution of grain size, EBSD.

Examinations were performed using the EBSD technique to confirm the phase composition of Docol 1300M steel and the precipitates observed in the SEM. Based on the approach, it was confirmed that Docol 1300M steel in the region of the base material is characterized by a ferritic microstructure (Figure 8) with a grain size in the range of about 0.5–10 µm (Figure 8c). A martensitic microstructure and titanium nitride precipitates of about 1–5 µm (Figure 9) were found in the welded zone. Based on the analysis of the results of inverse polar figures (Figure 9f), no texture was identified in the microstructure.

**Figure 10:**
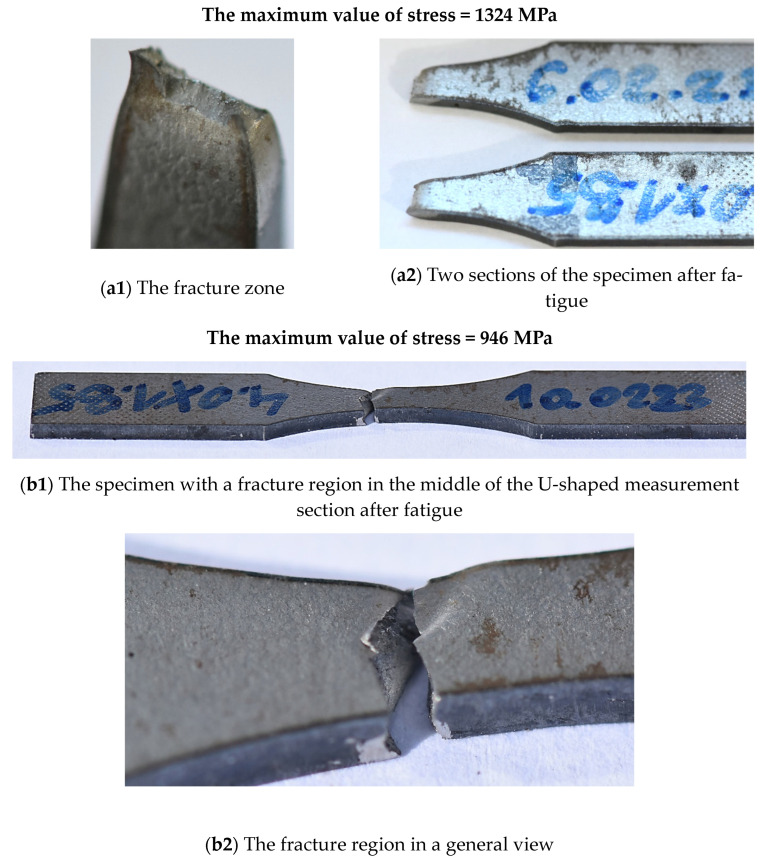

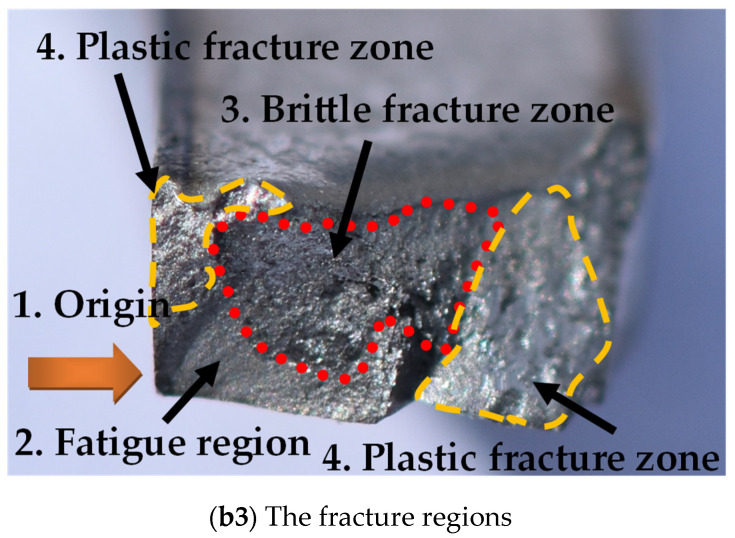
Fracture of the Docol 1300M weld due to fatigue under 1324 MPa and 946 MPa.

**Figure 11:**
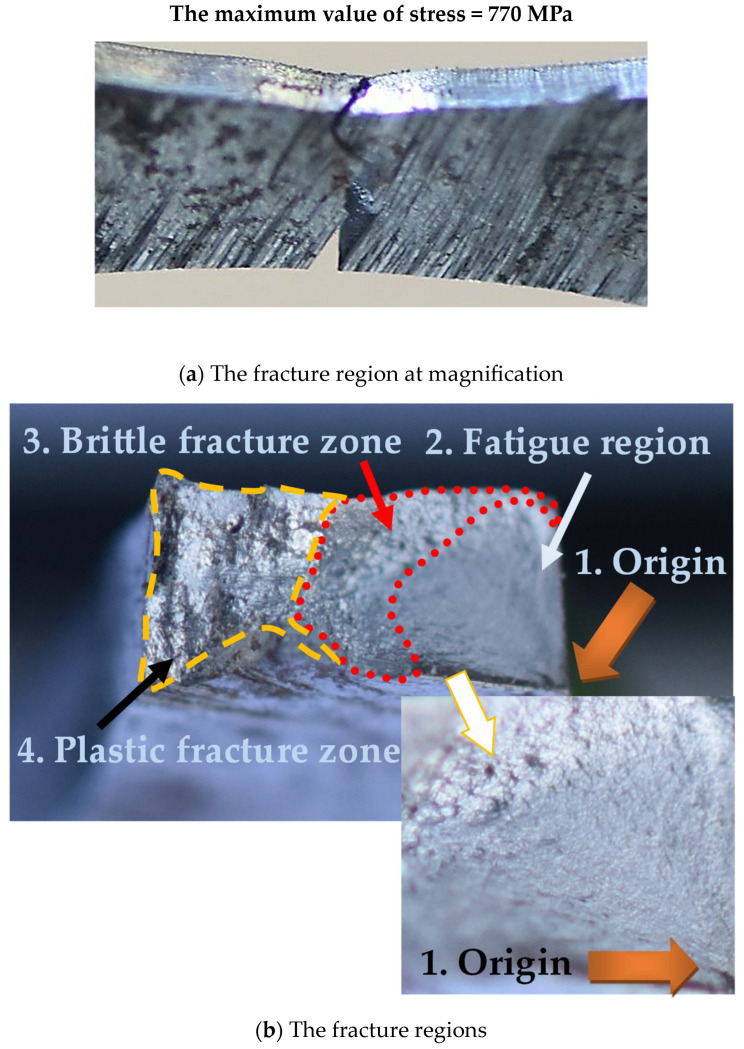
Fracture of the Docol 1300M weld due to fatigue under 770 MPa.

The weld fracturing was related to the maximum value of stress because differences in the joint degradation were visible. If the stress value reaches ultimate tensile strength, then a shear stress component is secondary in comparison to an axial one, which plays a main role in decohesion, leading to a single-plane fracturing at a significant necking (Figure 10(a1,a2)). This was not observed at a lower value of stress (946 MPa) (Figure 10(b1–b3)) when the fracture zone was represented by a more complicated shape and small necking, which indicates weld degradation at more complex stress components, i.e., axial and shear ones. This was also confirmed for the stress value equal to 770 MPa (207 MPa below the value of elastic limit) (Figure 11), but in this case, the role of shear stress was more significant. It is worth noticing that origins due to fatigue were not dependent on the stress levels used. This section occurred in the corner of the fracture area (Figure 10(b3) and Figure 11b). As can be observed in the case of a higher value of stress (Figure 10(b3)), the fatigue region covers a bigger area compared to the smaller one (Figure 11b). This is typical if stress values are different. 

**Figure 12:**
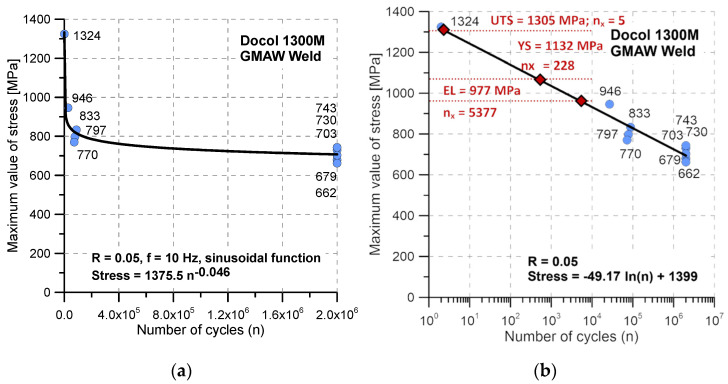
The Wöhler curve of the Docol 1300M weld (GMAW) steel determined at tension cycles for R = 0.05: (**a**) with fatigue limit and (**b**) with mechanical parameters of the steel and number of cycles to fracture.

Results of the tension cyclic test at R = 0.05 expressed a wide range of stress values, represented by the following levels: 1324 MPa and 662 MPa (Figure 12a). This indicates the steel can be operated at high-stress values up to 780 MPa covering 0.2 × 10^6^, and if the stress value is close to 707 MPa, then this kind of material reaches 2 × 10^6^. Taking the fatigue limit value with ultimate tensile strength, the proportion between both mechanical parameters can be proposed. For the Docol 1300M, this reached 1.85. Other details concerning an inspection can be found in Figure 12b, which illustrates the values of mechanical parameters and the connected number of cycles to fracture. This indicates the very narrow range of loading cycles for the stress value between the elastic limit and yield stress, represented by a value equal to only 4360.

## 4. Conclusions

The research novelty described in the article involves the simultaneous use of a newly developed mixture of shielding gas containing nitrogen along with micro-jet cooling. Neither argon shielding compounds with nitrogen nor as micro-jet cooling have been considered for AHSS steels so far. A new grade of steel DOCOL 1300M was used, which, according to the literature data, is characterized by a significant deterioration of mechanical properties after welding.

In the solution, aimed at significantly increasing the mechanical properties of the DOCOL 1300M steel joint, several entities were proposed:

- Micro-Jet cooling was used at constant parameters, which promoted the formation of a martensitic structure. This structure affected the mechanical properties of the weld, the hardness, and strength;
- Shielding gas mixtures with variable nitrogen content (0–2% N_2_) were used, which had an impact on the formation of nitrides reinforcing the weld.

The dual use of micro-jet cooling solutions and the supply of nitrogen to the weld through a protective mixture allowed for a significant increase in the mechanical properties of the Docol 1300M steel joint. The structure and mechanical properties of the joint were checked for different test variants. The joints and various accompanying phenomena were thoroughly examined, focusing primarily on observing the joint under a scanning microscope and testing the joint strength and fatigue strength. The best results were achieved for the Ar-1%N_2_ shielding gas mixture. This allows for an increase in the amount of nitride non-metallic inclusions (mainly TiN), which strengthen the weld. The Docol 1300M steel weld manufactured using gas metal arc welding yielded the joint with very attractive mechanical parameters for operation because these parameters were higher than the base metal, and the fatigue limit reached 707 MPa at tensile cycles.

The addition of nitrogen to argon must be carefully controlled because too high a nitrogen concentration may promote the growth of non-metallic inclusions, which may cause welding defects. The proposed welding technology with the Ar-1%N_2_ shielding gas mixture for the AHSS-grade steel can be directly used for manufacturing components recommended for various branches of industry. For future research in the welding ofDocol 1300 M steel, it is planned to test various micro-jet cooling parameters using a chosen shielding gas mixture containing 1% of nitrogen in the MAG process.

## Figures and Tables

**Table 1:** Chemical composition (wt. %) of the Docol 1300M.

Steel Grade	C (%)	Si (%)	Mn (%)	P (%)	S (%)	Al (%)	Nb (%)	Ti (%)
Docol 1300M	0.14	0.21	1.35	0.012	0.002	0.041	0.16	0.026

**Table 2:** Chemical composition (wt. %) of the electrode wire.

Electrode Wire Type	C (%)	Si (%)	Mn (%)	Cr (%)	Mo (%)	Ni (%)	Ti (%)
UNION X90	0.10	0.81	1.81	0.035	0.61	2.55	0.0068
UNION X96	0.11	0.83	1.78	0.002	0.48	2.46	0.0073

**Table 3:** The welding process parameters.

Number of Layers	Welding Method	Wire Diameter (mm)	Current (A)	Voltage (V)	Polarization	Welding Speed (mm/min)	Unit Energy (kJ/cm)
1	GMAW	1.0	114	20	DC “+”	370	3.3

**Table 4:** Results of NDT.

Electrode Wire	Shielding Gas	Specimen Code	Image	Observation Result
Union X90	Ar	PG1	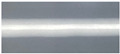	No cracks in the joint
Union X90	Ar-1% N_2_	PG2	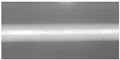	No cracks in the joint
Union X90	Ar-2% N_2_	PG3	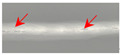	Small cracks in the joint
Union X96	Ar	PG4	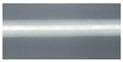	No cracks in the joint
Union X96	Ar-1% N_2_	PG5	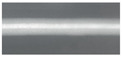	No cracks in the joint
Union X96	Ar-2% N_2_	PG6	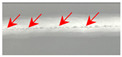	Cracks in the joint

**Table 5:** Values of Vickers, Brinell hardness, and ultimate tensile strength (UTS).

Specimen Code	Base Material			HAZ			Weld		
	HV (MPa)	HB (MPa)	UTS (MPa)	HV (MPa)	HB (MPa)	UTS (MPa)	HV (MPa)	HB (MPa)	UTS (MPa)
PG1	338	321	1107	352	335	1156	310	295	1018
PG2	337	320	1104	354	337	1163	314	299	1032
PG4	338	321	1107	354	337	1163	318	302	1042
PG5	337	320	1104	355	338	1166	325	309	1066

**Table 6:** Mechanical properties of Docol 1300M weld. E, Young’s modulus; PL, proportional limit; EL, elastic limit (at 0.05% plastic strain); YS, proof yield stress (at 0.2% plastic strain); UTS, ultimate tensile strength; RE, relative elongation.

E (MPa)	PL (MPa)	EL (MPa)	YS (MPa)	UTS (MPa)	RE (%)
2.2 × 10^5^	704	977	1132	1305	8

## Data Availability

The original contributions presented in the study are included in the article, further inquiries can be directed to the corresponding authors.
